# Editorial: Syphilis infection: clinical, epidemiology, basic science, and behavioral research

**DOI:** 10.3389/fimmu.2023.1182069

**Published:** 2023-04-06

**Authors:** Pingyu Zhou, Meiping Ye, Joseph D. Tucker, Lei Zhang, Justin David Radolf

**Affiliations:** ^1^ STD Institute, Shanghai Skin Disease Hospital, Tongji University School of Medicine, Shanghai, China; ^2^ Department of Dermatology, Xinhua Hospital, Shanghai Jiaotong University School of Medicine, Shanghai, China; ^3^ Departments of Medicine, Pediatrics, Genetics and Genomic Science, Molecular Biology and Biophysics, and Immunology, UConn Health, Farmington, CT, United States; ^4^ Artificial Intelligence and Modelling in Epidemiology Program, Melbourne Sexual Health Centre, Alfred Health, Melbourne, VIC, Australia; ^5^ Central Clinical School, Faculty of Medicine, Monash University, Melbourne, VIC, Australia; ^6^ China-Australia Joint Research Center for Infectious Diseases, School of Public Health, Xi’an Jiaotong University Health Science Center, Xi’an, China; ^7^ Institute of Global Health and Infectious Diseases, University of North Carolina at Chapel Hill, Chapel Hill, NC, United States

**Keywords:** syphilis, clinical research, epidemiology, basic science, behavioral research

Syphilis is an ancient sexually transmitted disease caused by the spirochete *Treponema pallidum* subspecies *pallidum* (*T. pallidum*). Over the past decade, syphilis incidence has increased in many countries. Untreated syphilis can lead to serious health problems, including blindness, neurocognitive disorders, cardiovascular injury, and adverse pregnancy outcomes. Syphilis research is urgently needed to decrease morbidity and mortality. This Frontiers Research Topic focuses on diverse aspects of syphilis infection. Its purpose was to expand our knowledge of syphilis epidemiology, clinical management, public health control measures, vaccine development, and basic science.

Estimates of the incidence trends and general impact on the healthcare system are essential for syphilis prevention strategies. Fu et al. estimated global syphilis incidence in partnerhisp with the Global Burden of Disease Group. They found substantial increases in syphilis among men and people in higher-income countries. SŠmit et al. estimated that the incidence of syphilis in Germany rose from 5/100,000 person-years to 6.2/100,000 persons over a three-year period, resulting in a minimal annual cost to the German healthcare system of €20,292,110. A study from Solaimalai et al. in Southern India found that syphilis prevalence increased from 0.5% in 2015 to 2.1% in 2020 among non-pregnant persons at a single site in southern India. These data highlight the need for additional public health interventions and strategies. Tucker et al. called for the application to syphilis of COVID-19 prevention and control strategies, including new diagnostic pathways (e.g., syphilis self-testing), contact tracing services, and public engagement. Routine anal self-examination may enhance syphilis detection and control, especially among men who have sex with men (MSM). Aung et al. conducted a longitudinal study examining adherence to weekly anal self-examinations among MSM and concluded that men adhered well to weekly anal self-examination. Tran et al. evaluated interventions that did not focus on increasing condom use or testing among MSM and found evidence that doxycycline post-exposure prophylaxis (PEP) reduces syphilis incidence.

Early diagnosis and treatment of syphilis are of great importance in determining disease prognosis. Ren et al. presented a case report of an atypical form of primary cutaneous syphilis without a chancre called Folmann Balanitis. This case underscores the diversity of clinical manifestations associated with syphilis infection. In addition, clinical diagnosis and treatment of neurosyphilis remain challenging. This issue also included two neurosyphilis reviews, one focused on China and one broader in scope. [Zhou et al., Du et al.]. Many patients treated for syphilis infection do not have a robust serological response based on changes in nontreponemal titers, but little is known about the serofast state. The serofast state is defined as the failure of nontreponemal titers to decline with disappearance of clinical symptoms after an appropriate follow-up period following treatment. Luo et al. found that serofast responses were more common among older adults compared to younger adults. Liu et al. examined rates of serological cure in patients with asymptomatic neurosyphilis. These reports underline the need for more research on neurosyphilis.

*T. pallidum* is an invasive bacterium that can evade the host immune response and persist for decades, hence its designation as “the stealth pathogen”. The Research Topic includes articles on bacterial-host interactions and vaccine development. Houston et al. investigated the possibility of antimicrobial peptides (AMPs) production as an unrecognized defense strategy used by *T. pallidum* during infection. They demonstrated that AMPs exhibit bacteriostatic and bactericidal activity against a panel of biologically relevant bacteria. In addition, AMPs can differentially regulate the expression of two pro-inflammatory chemokines - monocyte chemoattractant protein-1 (MCP-1) and interleukin-8 (IL-8). The finding that *T. pallidum* expresses AMPs to defend against competing microbes and modulate the host immune response could be important for its ability to establish infection following transmission. Li et al. reported the potential role of m6A methylation in syphilis pathophysiogenesis, and they provided evidence that YTHDF1 negatively regulates *T. pallidum*-induced inflammation in the THP-1 macrophage cell line by promoting socs3 translation in an m6a-dependent manner. The paper by Xu et al. aimed to elucidate the role of the outer membrane lipoprotein TP0136 in activation and aggregation of human platelets. Their results suggest that Tp0136 elicits these effects by downregulating PAR1 and triggering PAR1-dependent Gq and Gi pathway activation.

There is still much research needed in order to develop an effective syphilis vaccine. Kojima et al. reviewed current technologies and approaches towards a syphilis vaccine. Additionally, Molini et al. investigated B-cell epitopes of TprC and TprD variants and demonstrated that humoral responses are primarily directed to sequences predicted to be on surface-exposed loops of TprC and TprD proteins. This supports further exploration of TprC and TprD as vaccine candidates. This important research paves the way for further multi-disciplinary syphilis research.

This collection of papers has implications for policy, programs, and research. From a policy perspective, the costing analysis demonstrates the substantial cost of syphilis within health systems. The high prevalence of syphilis in many communities underscores that more resources are needed for syphilis control. From a program perspective, some of changes inspired by COVID-19 (e.g., greater attention to partner services and self-testing) may help to enhance syphilis programs. From a research perspective, further basic science and immunological research towards a vaccine are essential. A better understanding of syphilis pathogenesis could also enhance diagnostics.

## Author contributions

PZ, JT, LZ and JR contributed to the conceptualization and design of this editorial. MY drafted the manuscript. PZ, JT, LZ and JR revised it. All authors contributed to the article and approved the submitted version.

